# Associations of Overall Diet Quality and Individual Dietary Behaviours with Anthropometric Outcomes Among Chilean University Students

**DOI:** 10.3390/healthcare14182963

**Published:** 2026-09-11

**Authors:** Andrés Godoy-Cumillaf, Josivaldo de Souza-Lima, Maribel Parra-Saldias, Daniel Duclos-Bastias, Claudio Farias-Valenzuela, Eugenio Merellano-Navarro, José Bruneau-Chávez, Valentina Díaz-Goñi, Bruno Bizzozero-Peroni

**Affiliations:** 1 Grupo de Investigación en Educación Física, Salud y Calidad de Vida (EFISAL), Facultad de Educación, Universidad Autónoma de Chile, Temuco 4780000, Chile; andres.godoy@uautonoma.cl; 2 Facultad de Educación y Ciencias Sociales, Instituto del Deporte y Bienestar, Universidad Andres Bello, Las Condes, Santiago 7550000, Chile; josivaldo.desouza@unab.cl; 3 Departamento de Educación Física, Deporte y Recreación, Universidad de Atacama, Copiapó 1530000, Chile; maribel.parra@uda.cl; 4 iGEO, Escuela de Educación Física, Facultad de Filosofía y Educación, Pontificia Universidad Católica de Valparaíso, Viña del Mar 2340025, Chile; daniel.duclos@pucv.cl; 5 METIS Research Lab, Facultad de Negocios y Tecnología, Universidad Alfonso X el Sabio (UAX), 28691 Madrid, Spain; 6 Escuela de Ciencias de la Actividad Física, Universidad de Las Américas, Santiago 8320000, Chile; claudio.farias.valenzuela@edu.udla.cl; 7 Department of Physical Activity Sciences, Faculty of Education Sciences, Universidad Católica del Maule, Talca 3530000, Chile; emerellano@ucm.cl; 8 Departamento de Educación Física, Deportes y Recreación, Universidad de la Frontera, Temuco 4811230, Chile; jose.bruneau@ufrontera.cl; 9 Health and Social Research Center, Universidad de Castilla-La Mancha, 16002 Cuenca, Spain; bruno.bizzozero@uclm.es; 10 Faculty of Education, Universidad de Castilla-La Mancha, 13071 Ciudad Real, Spain; 11 Higher Institute of Physical Education, Universidad de la República, Maldonado 20000, Uruguay

**Keywords:** dietary habits, diet, adiposity, body mass index, waist circumference, network analysis

## Abstract

**Background/Objectives**: Diet quality is an important determinant of health during young adulthood; however, associations between dietary behaviours and anthropometric outcomes in university students remain insufficiently understood. This study examined the associations between dietary behaviours and anthropometric indicators in Chilean university students. **Methods**: A cross-sectional study was conducted among university students from southern Chile between June and November 2025. Dietary habits were assessed using a validated Chilean dietary habits questionnaire, from which Healthy Eating, Unhealthy Eating, and Overall Diet scores were derived. Anthropometric measurements included body mass index (BMI), waist circumference, and waist-to-height ratio (WHtR). Age- and sex-adjusted multivariable linear regression models were fitted. Exploratory analyses included psychometric evaluation, principal component analysis, false discovery rate (FDR) correction, and dietary behaviour network analysis. **Results**: A total of 374 students were included, of whom 40.3% were women. Psychometric analyses provided only partial support for the predefined two-domain structure, with stronger properties for the healthy domain. None of the predefined dietary scores were associated with BMI. The Unhealthy Eating Score was nominally positively associated with waist circumference (β = 0.285 cm per score point, 95% CI: 0.011–0.560), although this association did not remain statistically significant after FDR correction. Exploratory item-level analyses identified positive associations between sugar-sweetened beverage consumption and waist circumference and inverse associations between breakfast consumption and BMI after FDR correction. **Conclusions**: Predefined dietary behaviour scores showed limited associations with anthropometric outcomes. Psychometric analyses provided only partial support for the predefined scoring structure, particularly for the unhealthy dietary domain. Exploratory item-level analyses suggested that specific dietary behaviours may provide complementary information beyond that obtained by composite dietary scores, although these findings require confirmation in prospective longitudinal studies and further psychometric validation.

## 1. Introduction

Healthy dietary habits during young adulthood are fundamental determinants of both immediate health and long-term chronic disease risk. Late adolescence and early adulthood represent a critical transitional period characterized by increasing autonomy over food choices, exposure to new social and academic environments, and the establishment of lifestyle behaviours that frequently persist throughout adulthood [1,2]. Longitudinal cohort studies have shown that dietary patterns established during this life stage are associated with subsequent weight trajectories, cardiometabolic risk factors, and overall diet quality later in adulthood [3,4]. Consequently, promoting healthy dietary habits during young adulthood is widely recognized as an important strategy for the primordial prevention of noncommunicable diseases.

University students constitute a particularly vulnerable population for the adoption of unhealthy dietary behaviours because the transition to university is frequently accompanied by greater autonomy, academic stress, financial constraints, irregular meal patterns, and changes in the food environment [5,6]. Recent systematic reviews have reported poor diet quality, insufficient fruit and vegetable intake, and frequent consumption of sugar-sweetened beverages and fast food among university students across diverse geographic settings [7,8], with similar trends observed in Latin America and Chile [9,10]. The university food environment, often characterized by limited availability of healthy food options and widespread promotion of energy-dense products, may further reinforce these unhealthy eating behaviours [11]. These behaviours may have implications for anthropometric health, as healthier dietary patterns have generally been associated with lower BMI, waist circumference, and risk of overweight and obesity [12,13]. Because central adiposity is more closely related to cardiometabolic risk than is overall body mass alone, evaluating waist circumference and waist-to-height ratio alongside BMI may provide a more comprehensive assessment of diet-related anthropometric health [14,15,16].

From an epidemiological perspective, BMI and waist circumference should be considered complementary rather than interchangeable anthropometric indicators. BMI reflects overall body mass relative to height and remains the most widely used measure for population surveillance and clinical classification because of its simplicity, low cost, and standardized interpretation [17]. However, BMI does not distinguish between fat mass and lean mass nor does it provide information on body fat distribution, meaning that individuals with similar BMI values may exhibit substantially different cardiometabolic risk profiles [18,19]. In contrast, waist circumference and waist-to-height ratio serve as practical surrogate markers of central adiposity and have demonstrated greater sensitivity to dietary exposures in several recent population-based studies [20,21]. Consequently, specific dietary behaviours, including frequent consumption of sugar-sweetened beverages and inadequate fibre intake, may show stronger associations with abdominal adiposity than with overall body weight, reinforcing the importance of incorporating multiple anthropometric indicators when investigating diet-related health outcomes [22].

Validated dietary habit questionnaires provide a practical approach for assessing habitual eating behaviours in large populations, and composite scores can summarise overall dietary quality, while reducing the complexity of individual questionnaire responses [23,24,25,26]. However, composite scores may obscure associations involving specific dietary behaviours, making item-level analyses potentially informative as a complementary approach [27]. Moreover, because dietary behaviours are multidimensional, evaluating the measurement properties and underlying structure of these instruments is important for interpreting score-based epidemiological associations [28,29,30,31].

Despite important advances in dietary assessment methodology, several knowledge gaps remain. Although considerable effort has been devoted to validating dietary assessment instruments, and separately, to investigating associations between diet quality and anthropometric outcomes, comparatively fewer studies have integrated comprehensive psychometric evaluation with epidemiological analyses within the same study population [23,25,26,29]. Next, although predefined dietary scores are widely used to summarize overall diet quality, less attention has been paid to whether analyses of individual dietary behaviours provide complementary information beyond these composite indicators. Finally, evidence among Chilean university students remains limited. Although a dietary habits questionnaire has previously been developed and applied among Chilean university students [32], its psychometric properties in contemporary university populations and its associations with multiple anthropometric indicators have not been comprehensively examined using robust analytical approaches. Addressing these gaps is particularly relevant because BMI and indicators of central adiposity capture complementary dimensions of diet-related health risks in young adults [14].

To address these knowledge gaps, the present study integrates psychometric evaluation with epidemiological analyses to provide a comprehensive assessment of dietary habits and their associations with anthropometric indicators in Chilean university students. By combining predefined dietary scores with exploratory analyses of individual dietary behaviours, the study also examines whether these complementary analytical approaches provide additional insights into diet-related anthropometric health.

The aim of this study was to evaluate the psychometric properties of a validated dietary habits questionnaire and to investigate the associations of both predefined dietary scores and individual dietary behaviours with anthropometric indicators in Chilean university students. We hypothesized that healthier dietary behaviours would be associated with more favourable anthropometric indicators and that analyses of individual dietary behaviours would provide complementary information beyond that obtained from composite dietary scores.

## 2. Materials and Methods

### 2.1. Study Design and Participants

This cross-sectional observational study was conducted among undergraduate students from multiple academic programmes at the Universidad Autónoma de Chile, Temuco campus, Chile, between June and November 2025. Participants were recruited using a convenience sampling strategy through institutional e-mails, social media advertisements, and informational posters displayed across the university campus. Students who voluntarily agreed to participate and met the eligibility criteria were enrolled in the study.

Participants were undergraduate students enrolled in eight degree programmes across three faculties. These included Kinesiology and Chemistry, as well as Pharmacy, within the Faculty of Health Sciences; Psychology and Public Administration within the Faculty of Social Sciences and Humanities; and Primary Education, Early Childhood Education, Physical Education, and English Pedagogy within the Faculty of Education. Participants were predominantly enrolled in the first or second academic year, with Physical Education students from the first, second, and third academic years included.

Anthropometric measurements and dietary assessments were performed by the principal investigator and a team of trained health professionals who received standardized instruction before data collection to ensure consistency in measurement procedures and questionnaire administration.

The study was conducted in accordance with the ethical principles established in the Declaration of Helsinki. Ethical approval was obtained from the Ethics Committee of the Universidad Autónoma de Chile (protocol code: CEC 28-25; date of approval: 3 June 2025), and all participants signed an informed consent document before participation.

### 2.2. Eligibility Criteria

Eligible participants were undergraduate students aged ≥18 years enrolled at the Universidad Autónoma de Chile, Temuco campus, who voluntarily agreed to participate and provided written informed consent. Participants were excluded if they were pregnant, had medical conditions that could compromise the accuracy of anthropometric measurements, or lacked the information required for the corresponding analyses.

### 2.3. Sample Size

No formal a priori sample size calculation was performed. Participants were recruited using a convenience sampling strategy during the predefined study period. Of 520 students invited to join, 452 agreed to participate, 415 completed the study measurements, and 374 were included in the final analytical sample.

### 2.4. Anthropometric Assessment

Body weight, height, and waist circumference were assessed by the principal investigator and trained health professionals following standardized anthropometric protocols to minimize inter-observer variability. Participants were evaluated wearing light clothing and without shoes.

Body weight was measured to the nearest 0.1 kg using a calibrated digital scale (Tanita MC 780U). Standing height was measured to the nearest 0.1 cm using a portable stadiometer (Seca Model 200), with participants’ heads positioned in the Frankfurt plane. Body mass index (BMI) was subsequently calculated as body weight divided by the square of height (kg/m^2^).

Waist circumference was measured using a non-elastic anthropometric tape (Holway) at the midpoint between the lower margin of the last palpable rib and the iliac crest at the end of a normal expiration. Waist-to-height ratio (WHtR) was calculated by dividing waist circumference (cm) by height (cm).

### 2.5. Dietary Habits Assessment

Dietary habits were assessed using the dietary habits questionnaire developed and validated by Durán et al. [32] for the Chilean university population. The questionnaire assesses the usual frequency of selected food consumption and eating behaviours commonly observed among young adults. It has been widely used in epidemiological studies involving Chilean university students [33,34,35,36].

The instrument comprises 15 items grouped into two conceptual domains: healthy dietary habits (nine items) and unhealthy dietary habits (six items). The healthy dietary habits domain includes behaviours generally recommended in national and international dietary guidelines, such as regular consumption of fruits, vegetables, legumes, dairy products, fish, whole grains, and breakfast. In contrast, the unhealthy dietary habits domain captures behaviours typically associated with poorer diet quality, including frequent consumption of sugar-sweetened beverages, fried foods, fast food, pastries and sweets, processed snacks, and the addition of salt to meals. Each item is scored using a five-point Likert-type scale reflecting the frequency of the corresponding dietary behaviour, with higher scores indicating greater frequency of the corresponding behaviour.

For the primary analyses, three dietary indicators were calculated. The Healthy Eating Score was obtained by summing the nine healthy-habit items (possible range: 9–45), with higher values indicating greater adherence to healthy dietary behaviours. The Unhealthy Eating Score was calculated as the sum of the six unhealthy-habit items (possible range: 6–30), with higher values reflecting more frequent unhealthy dietary behaviours. An Overall Diet Score was computed by subtracting the Unhealthy Eating Score from the Healthy Eating Score, with higher values representing a more favourable overall dietary profile.

The questionnaire was administered face-to-face by the principal investigator and trained research staff. Standardized instructions were provided before administration, and participants were encouraged to request clarification whenever necessary to ensure accurate interpretation of all questionnaire items.

In addition to the predefined primary analyses based on the questionnaire scores, several complementary exploratory analyses were performed to obtain a more comprehensive understanding of dietary behaviours. Principal component analysis (PCA) was used to identify empirical dietary patterns based on the questionnaire items, while individual questionnaire items were examined to determine whether item-level analyses provided complementary information regarding anthropometric outcomes beyond that obtained by the predefined dietary scores. These exploratory approaches were intended to complement rather than replace the conventional score-based analyses and were not designed to formally quantify incremental predictive value beyond that obtained from the predefined dietary scores. Rather, they were used to identify behaviour-specific associations that might not be fully captured by composite dietary indices.

### 2.6. Data Management and Quality Control

Before statistical analyses, the dataset underwent a structured quality-control process to ensure data integrity and analytical consistency. Data screening included verification of variable formats, identification of duplicate records, assessment of missing values, and evaluation of implausible anthropometric measurements.

Potential inconsistencies in anthropometric variables were examined through internal cross-validation using body weight, height, body mass index, waist circumference, and waist-to-height ratio. When data-entry inconsistencies were identified, values were reviewed and reconstructed only when internal consistency among the recorded variables allowed unequivocal verification. Anthropometric values that could not be reliably verified were considered incomplete during the data-cleaning process and were considered when defining the final analytical sample.

Duplicate records were identified through comparison of participant information and questionnaire responses. When exact duplicate observations were detected, only the first complete record was retained for analysis. Of the 415 participants who completed the study measurements, 41 (9.9%) were excluded because of incomplete questionnaire and/or anthropometric data, resulting in a final analytical sample of 374 participants. Complete data were available for all main dietary and anthropometric variables among participants included in the analytical sample (0% missing data). Accordingly, complete-case analyses were performed, and no missing-data imputation was required.

All data-management procedures were completed before the statistical analyses, and no subsequent modifications were made to the analytical dataset.

### 2.7. Statistical Analysis

Statistical analyses were conducted using R statistical software version 4.5.1. (R Foundation for Statistical Computing, Vienna, Austria), whereas the exploratory dietary network analysis was performed using Python (version 3.13.13, Python Software Foundation, Beaverton, OR, USA) with the NetworkX library [37]. A two-sided *p*-value < 0.05 was considered statistically significant unless otherwise specified.

Continuous variables were summarized as means and standard deviations (SD) or as medians and interquartile ranges (IQR), according to their distribution, whereas categorical variables were expressed as frequencies and percentages. Distributional assumptions were evaluated using the Shapiro–Wilk test, together with graphical inspection of histograms and Q–Q plots. Differences between men and women were assessed using independent-samples *t*-tests or Mann–Whitney U tests for continuous variables and χ^2^ tests for categorical variables, as appropriate.

The psychometric properties of the dietary habits questionnaire were evaluated using Cronbach’s alpha and McDonald’s omega to assess internal consistency. The underlying questionnaire structure was examined through exploratory factor analysis after assessing sampling adequacy using the Kaiser–Meyer–Olkin statistic and Bartlett’s test of sphericity. The number of retained factors was determined using parallel analysis [38]. Confirmatory factor analysis was used to evaluate the fit of the predefined two-domain structure underlying the Healthy and Unhealthy Eating Scores, using the comparative fit index (CFI), the Tucker–Lewis index (TLI), the root mean square error of approximation (RMSEA), and the standardized root mean square residual (SRMR). These psychometric analyses were intended to evaluate the predefined scoring structure in the present sample rather than to redefine the primary dietary exposures. PCA was additionally performed as a separate exploratory dimensionality-reduction analysis to explore empirical dietary patterns across questionnaire items [39,40].

Primary associations between dietary habits and anthropometric outcomes were evaluated using multivariable linear regression models adjusted for age and sex. Healthy Eating Score, Unhealthy Eating Score, and Overall Diet Score were examined as the primary dietary exposures, whereas body mass index (BMI), waist circumference and waist-to-height ratio were analysed as dependent variables.

Residual diagnostics showed heteroscedasticity and departures from normality. Therefore, HC3 heteroscedasticity-consistent standard errors were used to obtain robust confidence intervals and statistical tests [41]. Model assumptions were evaluated through residual diagnostics, multicollinearity assessment using variance inflation factors, and influence diagnostics based on Cook’s distance. The robustness of the primary findings was further examined using robust regression models and bootstrap resampling with 5000 iterations. Age and sex were selected a priori as covariates because of their established relationships with both dietary behaviours and anthropometric characteristics rather than through a data-driven selection procedure. Other potentially relevant behavioural and socioeconomic covariates were not collected and therefore, could not be included in the primary models.

Additional exploratory analyses were conducted to further investigate the relationship between dietary behaviours and anthropometric outcomes. These included multivariable regression models using individual questionnaire items, false discovery rate (FDR) correction according to the Benjamini–Hochberg procedure [42], and an exploratory weighted undirected dietary-behaviour network based on pairwise Spearman rank correlations among the 15 questionnaire items [43]. An absolute Spearman correlation coefficient ≥ 0.12 was selected pragmatically to exclude very weak pairwise correlations and improve network interpretability. Thresholding of weak correlations is commonly used in correlation-network construction, although no consensus exists regarding an optimal absolute threshold [44]. Dietary behaviour communities were subsequently identified using the Clauset–Newman–Moore greedy modularity maximization algorithm [45]. Node strength centrality was calculated to characterise the relative importance of individual dietary behaviours [46], whereas one-step expected influence was additionally estimated to preserve the direction of positive and negative associations within the network [47]. These analyses were considered exploratory and were performed to identify potential behaviour-specific associations that might not be fully captured by the predefined dietary scores. Accordingly, findings derived from these analyses were interpreted as hypothesis-generating rather than confirmatory.

## 3. Results

Of 520 students invited to join, 452 agreed to participate (86.9%), of whom 415 completed the study measurements. Forty-one participants were excluded because of incomplete questionnaire and/or anthropometric data. A total of 374 university students were included in the final analyses, of whom 40.3% were women. The descriptive characteristics of the study population, according to sex, are presented in Table 1.

Overall, women presented significantly higher Overall Diet Scores than did men, whereas height was slightly greater in men. No significant sex-related differences were observed for age, body weight, BMI, waist circumference, waist-to-height ratio, Healthy Eating Score, or Unhealthy Eating Score.

Given the multidimensional nature of the dietary questionnaire, its psychometric properties and underlying factor structure were subsequently examined.

The psychometric evaluation of the dietary habits questionnaire yielded mixed findings (Table 2). Internal consistency was moderate for the Healthy Eating Score (Cronbach’s α = 0.670), whereas low internal consistency was observed for the Unhealthy Eating Score (α = 0.472) and the Overall Diet Score (α = 0.516).

The exploratory factor and parallel analyses suggested a three-factor structure, indicating that the empirical dimensionality of the questionnaire did not fully reproduce the predefined two-domain scoring system.

**Figure 1:**
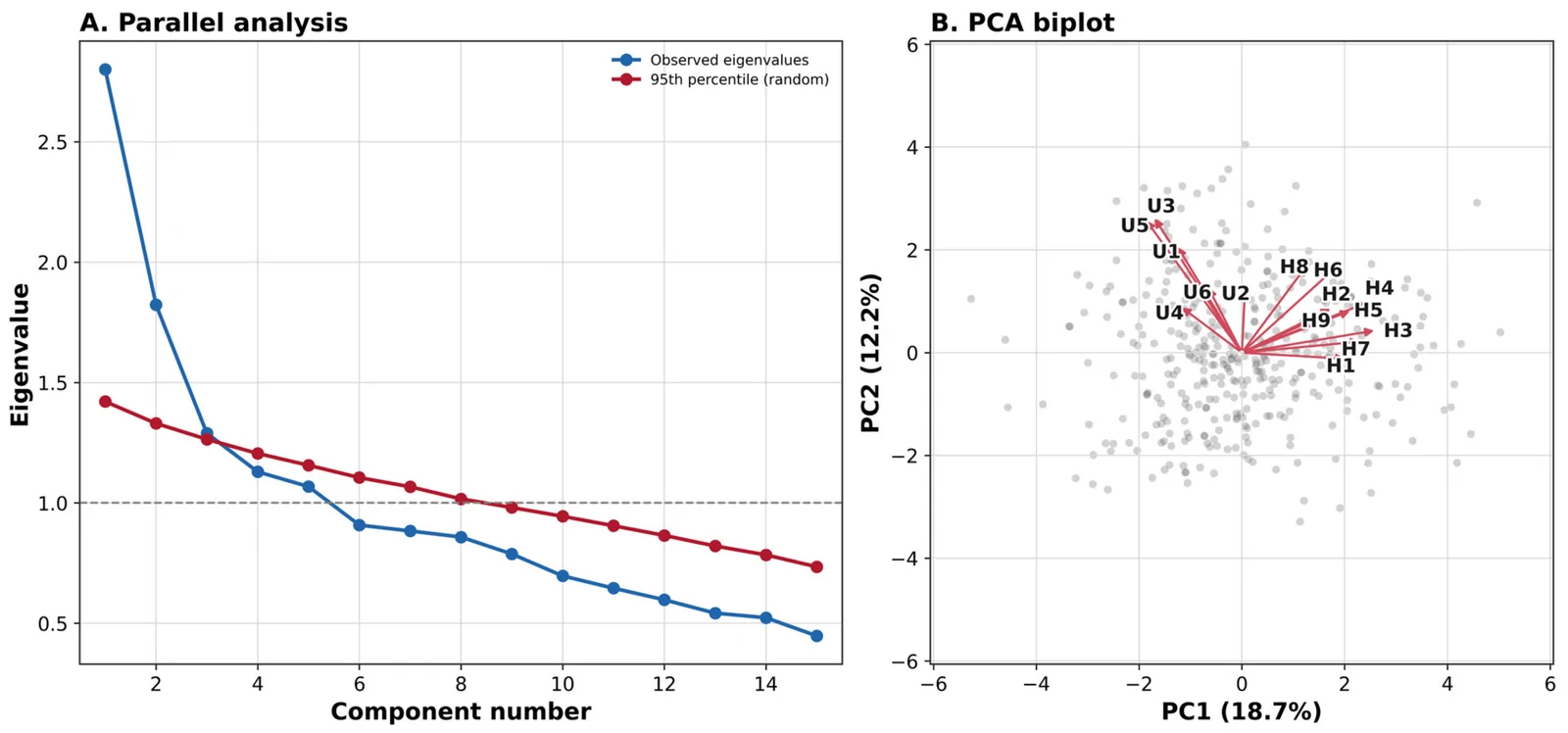
Parallel analysis and principal component analysis of the dietary habits questionnaire. (**A**) Parallel analysis comparing observed and randomly generated eigenvalues to determine the number of retained components. (**B**) Principal component analysis biplot illustrating the loading of individual questionnaire items on the first two principal components. Item codes are as follows: H1, snack consumption; H2, low-fat dairy consumption; H3, fruit consumption; H4, vegetable consumption; H5, fish consumption; H6, legume consumption; H7, oat or whole-grain bread consumption; H8, home-cooked meal consumption; H9, dinner consumption; U1, alcoholic beverage consumption; U2, sugar-sweetened beverage consumption; U3, fried food consumption; U4, adding salt to food before tasting; U5, fast-food consumption; U6, breakfast consumption.

Exploratory factor analysis supported the suitability of the questionnaire for dimensionality reduction, with an adequate Kaiser–Meyer–Olkin measure and a highly significant Bartlett’s test of sphericity. Parallel analysis suggested retention of three factors rather than of the two domains underlying the predefined Healthy and Unhealthy Eating Scores (Figure 1A). Confirmatory factor analysis of the predefined two-domain structure yielded mixed evidence of model fit (Table 2). Although RMSEA and SRMR values were within commonly accepted ranges, CFI and TLI were below conventional thresholds, providing only partial support for the predefined scoring structure. PCA was examined separately as an exploratory dimensionality-reduction approach rather than as a validation of the predefined scoring system (Figure 1B). Inspection of the PCA loading pattern suggested that the first principal component largely reflected a gradient from healthier to less healthy dietary behaviours, whereas the second component captured additional variation related to specific eating habits. Together, the psychometric findings suggest that the empirical structure of dietary behaviours evaluated in this sample was more complex than was the predefined two-domain scoring system. Accordingly, the predefined scores were retained for the primary analyses because they represented the a priori scoring approach of the questionnaire, but their associations with anthropometric outcomes should be interpreted cautiously, particularly given the low internal consistency of the Unhealthy Eating Score.

**Figure 2:**
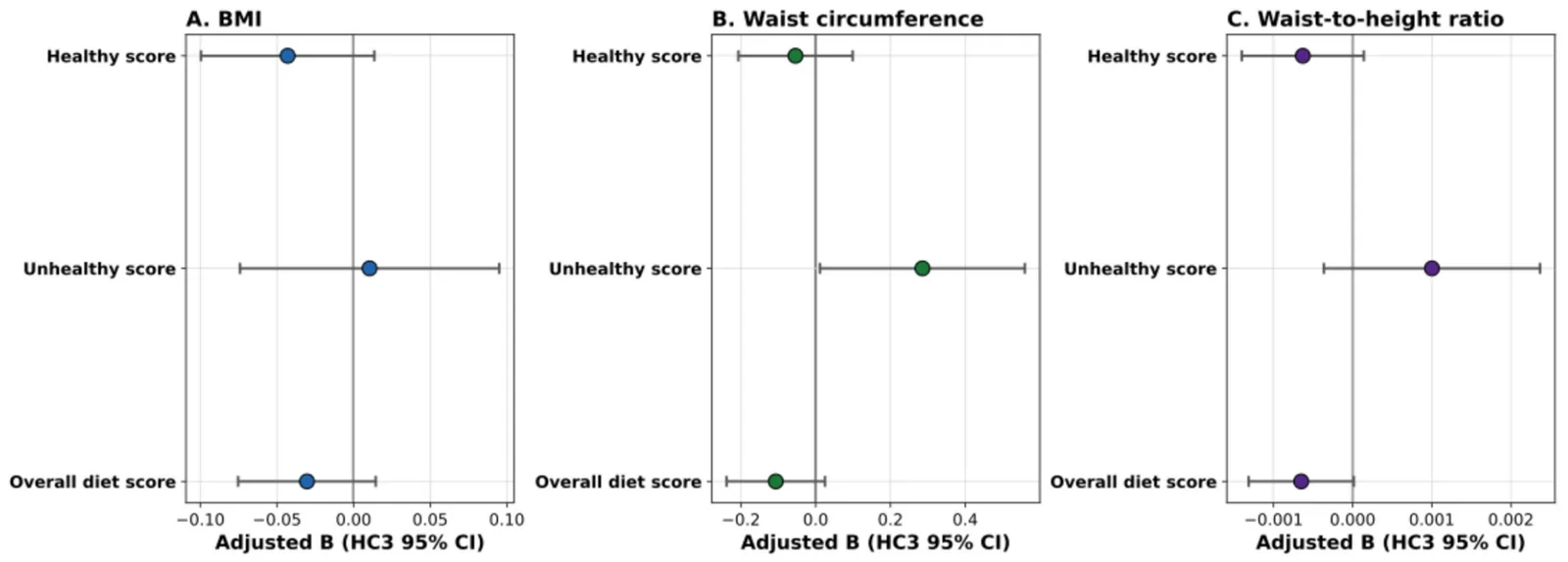
Associations between dietary scores and anthropometric outcomes. Regression coefficients and 95% confidence intervals based on HC3 heteroscedasticity-consistent standard errors from multivariable linear regression models adjusted for age and sex.

Overall, the predefined dietary scores showed limited associations with the evaluated anthropometric outcomes (Table 3). After adjustment for age and sex, none of the predefined dietary scores were significantly associated with BMI or WHtR. Similarly, neither the Healthy Eating Score nor the Overall Diet Score was significantly associated with waist circumference. Only the Unhealthy Eating Score was nominally positively associated with waist circumference (β = 0.285, 95% CI: 0.011 to 0.560; *p* = 0.041), although this association did not remain statistically significant after FDR correction. Figure 2 illustrates the regression coefficients and their corresponding 95% confidence intervals, showing that most confidence intervals crossed the null value, indicating limited evidence of associations between the global dietary scores and the evaluated anthropometric outcomes.

Sensitivity analyses using complementary robust analytical approaches yielded broadly similar effect estimates across body mass index (BMI), waist circumference, and waist-to-height ratio (WHtR), supporting the overall robustness of the primary findings (Supplementary Table S1). The direction and magnitude of the regression coefficients remained largely consistent across the sensitivity analyses, and no additional meaningful associations were identified.

Taken together, these findings indicate limited evidence of associations between the predefined dietary scores and anthropometric outcomes. The nominal association between the Unhealthy Eating Score and waist circumference did not remain statistically significant after FDR correction.

**Figure 3:**
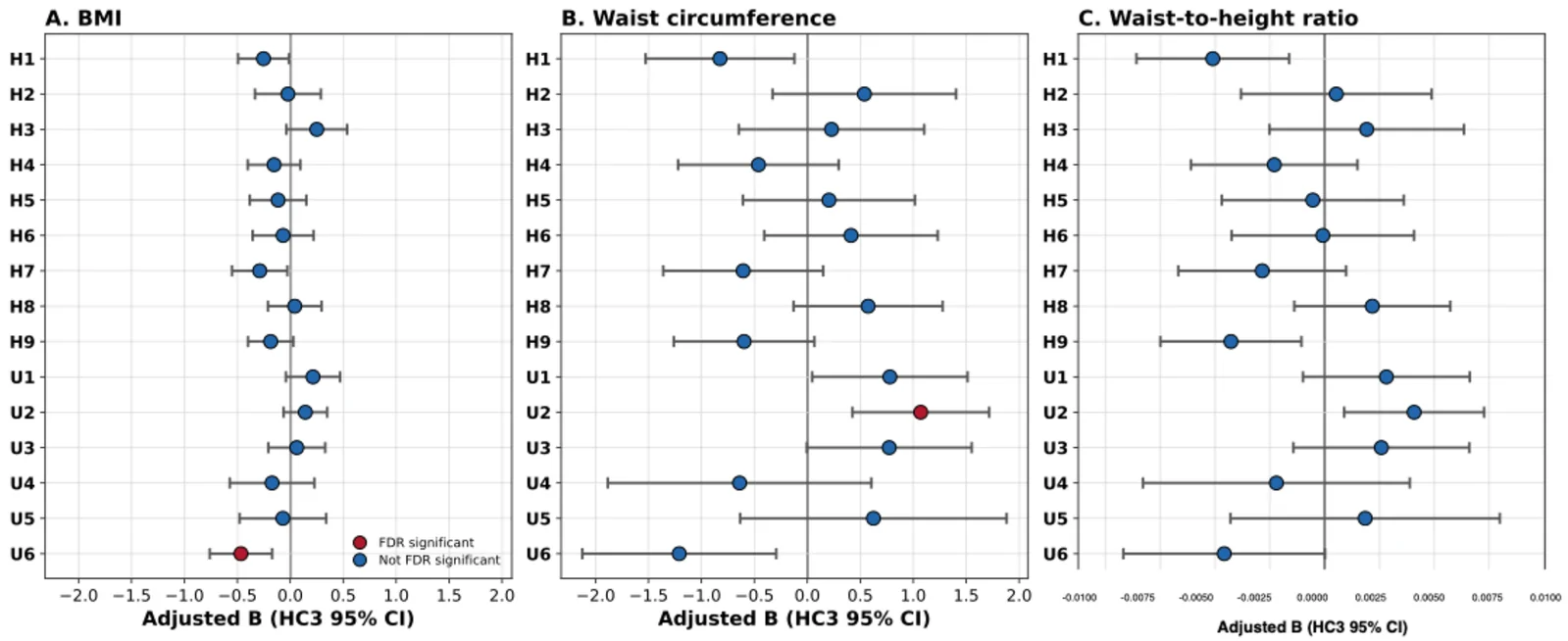
Associations between individual dietary questionnaire items and anthropometric outcomes. Forest plots showing regression coefficients and confidence intervals based on HC3 heteroscedasticity-consistent standard errors from models adjusted for age and sex. Red points indicate associations remaining statistically significant after false discovery rate correction. Item codes are as follows: H1, snack consumption; H2, low-fat dairy consumption; H3, fruit consumption; H4, vegetable consumption; H5, fish consumption; H6, legume consumption; H7, oat or whole-grain bread consumption; H8, home-cooked meal consumption; H9, dinner consumption; U1, alcoholic beverage consumption; U2, sugar-sweetened beverage consumption; U3, fried food consumption; U4, adding salt to food before tasting; U5, fast-food consumption; U6, breakfast consumption.

Exploratory item-level analyses were subsequently conducted to examine whether individual dietary behaviours showed associations that were not apparent in the predefined dietary score analyses (Figure 3 and Supplementary Table S2). Although most questionnaire items were not significantly associated with the evaluated anthropometric outcomes, several individual dietary behaviours showed associations that were not detected in the analyses based on the composite dietary scores.

After adjustment for age and sex and correction for multiple comparisons using the false discovery rate (FDR) procedure, only two dietary behaviours remained significantly associated with the evaluated anthropometric outcomes (Supplementary Table S2). More frequent consumption of sugar-sweetened beverages was positively associated with waist circumference, whereas more frequent breakfast consumption was inversely associated with BMI. No individual dietary behaviours remained significantly associated with waist-to-height ratio after FDR correction.

Taken together, these exploratory findings suggest that when examining associations with anthropometric indicators, specific dietary behaviours may provide complementary information beyond that obtained from global dietary scores. Separately, exploratory network analysis identified several highly interconnected dietary behaviours within the overall dietary network (Supplementary Table S3). However, because these analyses were exploratory in nature, the observed associations should be interpreted with caution and confirmed in future studies. These exploratory findings informed the interpretation of the primary analyses and were considered hypothesis-generating rather than confirmatory.

## 4. Discussion

This study investigated the associations between dietary habits, individual dietary behaviours, empirically derived dietary patterns, and anthropometric indicators in a sample of Chilean university students. Four principal findings emerged. First, the psychometric evaluation yielded mixed findings. Measurement properties were stronger for the healthy dietary domain, whereas internal consistency was comparatively low for the unhealthy domain. Second, predefined dietary scores showed only modest associations with anthropometric outcomes, with the Unhealthy Eating Score being nominally positively associated with waist circumference after adjustment for age and sex, whereas no meaningful associations were observed with BMI or waist-to-height ratio. Third, item-level analyses indicated that specific dietary behaviours, particularly sugar-sweetened beverage consumption in relation to waist circumference and breakfast-related behaviour in relation to BMI, provided complementary information beyond that captured by composite dietary scores. Finally, exploratory multivariate analyses, including PCA and network analysis, further supported the multidimensional nature of dietary behaviours, while item-level analyses identified modest and behaviour-specific associations with anthropometric outcomes.

The present study yielded mixed evidence regarding the psychometric performance of the dietary habits’ questionnaire for Chilean university students. Although the KMO measure and Bartlett’s test supported the suitability of the data for factor analysis, parallel analysis suggested a three-factor structure, which did not fully reproduce the predefined Healthy and Unhealthy Eating domains. Consistently, CFA provided only partial support for the predefined two-domain structure, with acceptable RMSEA and SRMR values but CFI and TLI values below conventional thresholds. PCA, considered separately as an exploratory dimensionality-reduction approach, further illustrated the multidimensional nature of the dietary behaviours assessed. These findings indicate that the questionnaire may provide a pragmatic assessment of selected habitual dietary behaviours, while the predefined scores should be regarded as a priori summary measures rather than as fully confirmed latent constructs in the present sample [47,48]. Accordingly, the relatively low reliability of the Unhealthy Eating Score should be considered when interpreting its associations with anthropometric outcomes.

These findings also explain the moderate internal consistency observed. Food choice, meal timing, beverage consumption, breakfast habits, snacking, cooking practices, and fruit and vegetable intake are influenced by distinct biological, behavioural, social, and environmental determinants and should not necessarily be expected to cluster within a single internally consistent construct. Accordingly, moderate Cronbach’s alpha values may be understandable in behavioural nutrition questionnaires designed to capture diverse dietary domains rather than repeated indicators of a single latent trait. In this context, excessively high internal consistency may even indicate item redundancy rather than broader coverage of relevant dietary behaviours [49,50].

The lower internal consistency observed for the unhealthy subscale should therefore not be interpreted in isolation as evidence of poor validity but rather as potentially reflecting the heterogeneous nature of unhealthy dietary behaviours within this university population. Behaviours such as frequent consumption of sugar-sweetened beverages, irregular breakfast habits, fast-food intake, and low consumption of health-promoting foods may occur in different combinations depending on students’ academic schedules, economic resources, food environments, living arrangements, stress levels, and social contexts. The multidimensional nature of dietary behaviour assessment has also been reported in Chilean university populations. Díaz et al. [51] adapted and validated a dietary habits questionnaire comprising 35 items distributed across 10 dimensions and reported acceptable overall internal consistency (Cronbach’s α = 0.815). Similarly, de Carvalho et al. [52] found that, among university students, a six-factor solution provided a better representation of eating behaviours than did the original three-factor model, further supporting the complexity and multidimensionality of dietary behaviour assessment in this population.

A higher Unhealthy Eating Score was nominally associated with greater waist circumference, whereas associations with BMI and waist-to-height ratio were weak or absent. This pattern may be biologically and epidemiologically plausible because waist circumference is a more direct indicator of abdominal adiposity and indirectly, visceral fat accumulation, whereas BMI reflects body mass relative to height, without distinguishing fat mass, lean mass, bone mass, or fat distribution. This may partly explain why the nominal association was more apparent for waist circumference than for BMI in this sample [53]. However, the association did not remain statistically significant after FDR correction and should therefore be interpreted cautiously. Moreover, the cross-sectional design precludes establishing the temporal relationship between dietary behaviours and anthropometric outcomes.

The nominal association observed for waist circumference may also reflect the greater metabolic sensitivity of central adiposity to dietary behaviours. Frequent consumption of energy-dense foods, sugar-sweetened beverages, highly processed snacks, and irregular meals has been associated with positive energy balance, impaired satiety regulation, chronic low-grade inflammation, and metabolic alterations that may favour abdominal fat accumulation before substantial increases in total body weight occur [54]. Nevertheless, the modest magnitude of the observed association is unsurprising, given that abdominal adiposity is influenced by multiple behavioural, biological, environmental, and genetic determinants, including physical activity, sedentary behaviour, sleep, psychosocial stress, alcohol consumption, socioeconomic conditions, and total energy intake. Recent evidence in young adults further highlights the interrelationships between nutritional and sleep behaviours and somatic health indicators, including sex-specific patterns [55]. Dietary habits therefore represent one component within a much broader network of biological, behavioural, environmental, and socioeconomic factors contributing to central fat accumulation [56,57].

Overall, the existing literature supports an association between poorer dietary quality and greater adiposity, although the magnitude and consistency of these associations vary considerably across populations and study designs. The 2025 Dietary Guidelines Advisory Committee systematic review concluded that healthier dietary patterns are generally associated with lower BMI, waist circumference, and risk of overweight or obesity, while less favourable dietary patterns show the opposite trend. However, the report also highlighted substantial heterogeneity in dietary pattern definitions, scoring methods, and dietary assessment instruments [57]. This heterogeneity is particularly relevant because the present questionnaire assessed habitual dietary behaviours rather than quantitative dietary intake, portion sizes, nutrient intake, or total energy intake. Accordingly, the relatively modest associations observed here should be interpreted within the context of the dietary assessment method rather than as evidence inconsistent with previous research. This interpretation is consistent with recent studies in young adults, which have likewise highlighted that associations between dietary quality and health-related outcomes may vary according to the dietary assessment methods employed and the characteristics of the study population [58].

Likewise, the absence of a robust association between global dietary scores and BMI should not be interpreted as evidence that diet is unrelated to body weight or adiposity. BMI is an imperfect indicator of adiposity, particularly among young adults with heterogeneous body composition and physical activity levels. Importantly, individuals with BMI values within the normal range may still present excess body fat, a phenotype commonly described as normal-weight obesity. Recent evidence in young adults has shown that this phenotype may coexist with greater visceral adiposity and lower lean body mass despite a normal BMI [59]. Brief dietary behaviour questionnaires are not designed to quantify portion sizes, total energy intake, nutrient composition, alcohol consumption, or compensatory eating behaviours [49,54,57]. These methodological considerations may attenuate the strength of observed associations, particularly in cross-sectional studies relying on behavioural dietary measures rather than on comprehensive dietary intake assessments. In addition, reverse causation cannot be excluded, as students with higher body weight may have already modified their dietary habits, and the relatively young age of the participants may limit the cumulative impact of dietary exposures on overall body mass.

Consistent with this interpretation, studies conducted among university students have reported heterogeneous associations between dietary behaviours and anthropometric outcomes. Recent systematic reviews indicate that university students frequently exhibit the coexistence of unhealthy dietary behaviours, irregular meal timing, low fruit and vegetable consumption, insufficient physical activity, and varying levels of overweight and obesity, although the strength of these associations differs considerably across populations and assessment methods [60]. Similarly, observational studies conducted in university populations have shown that healthier dietary and lifestyle behaviours are associated with more favourable anthropometric profiles, although these relationships are generally modest and are influenced by demographic and contextual factors [61]. Together, these findings suggest that the relatively modest score-based associations observed in the present study are consistent with the complexity of evaluating anthropometric outcomes using self-reported behavioural dietary measures in young and heterogeneous university populations. Because adiposity is influenced by multiple interacting behavioural, environmental, socioeconomic, and biological determinants, relatively modest associations between dietary behaviour scores and anthropometric indicators should be expected in cross-sectional analyses relying on brief dietary assessment instruments.

The item-level analyses suggested that individual dietary behaviours may provide complementary information beyond that obtained from composite dietary scores. Sugar-sweetened beverage consumption remained positively associated with waist circumference after controlling for multiple comparisons, whereas the breakfast-related item was associated with BMI. Although these findings should be considered exploratory and hypothesis-generating, they suggest that analysing individual dietary behaviours may reveal associations that become diluted when behaviours are aggregated into global dietary scores. This observation is particularly relevant because composite indices are designed to summarise overall dietary quality but may mask the contribution of specific behaviours that have stronger relationships with anthropometric outcomes. Rather than representing competing analytical strategies, global dietary scores and individual dietary behaviour analyses appear to capture different, yet complementary, dimensions of dietary exposure [48,62].

The positive association between sugar-sweetened beverage consumption and waist circumference is consistent with a substantial body of epidemiological evidence. Sugar-sweetened beverages provide rapidly absorbed liquid calories that elicit relatively weak satiety responses, potentially increasing total daily energy intake without equivalent reductions in subsequent food consumption. Moreover, frequent consumption of these beverages often occurs within broader unhealthy dietary patterns characterised by greater intake of ultra-processed foods, frequent snacking, lower dietary fibre intake, and irregular eating behaviours. Accordingly, sugar-sweetened beverage consumption may represent both a direct dietary exposure and a marker of an obesogenic lifestyle. Nevertheless, these findings should not be interpreted as evidence of a direct causal relationship, as beverage choices are also shaped by food availability, affordability, commuting time, academic workload, sleep deprivation, stress, social environments, and marketing exposure [63,64,65,66].

The association observed for the breakfast-related item requires even more cautious interpretation. Breakfast consumption is frequently regarded as a marker of dietary regularity and healthier daily routines rather than as an isolated protective behaviour, and evidence regarding its relationship with obesity remains inconsistent. Previous systematic reviews and meta-analyses have reported limited or no consistent evidence that breakfast-skipping independently influences BMI or waist circumference [67,68,69]. Therefore, the association identified in the present study should be interpreted as hypothesis-generating rather than confirmatory. Rather than representing an isolated dietary behaviour, breakfast habits likely reflect broader behavioural profiles encompassing meal timing, dietary quality, sleep patterns, physical activity, and socioeconomic circumstances. Reverse causation should also be considered, as students concerned about their body weight may intentionally modify breakfast habits following dietary advice or weight-control efforts.

The complementary exploratory network analysis provided additional insight into the interrelationships among dietary behaviours, with several behaviours occupying relatively more central positions within the dietary network. Taken together, these findings suggest that global dietary scores and item-level analyses should be viewed as complementary rather than competing approaches. While composite indices remain valuable for summarising overall dietary quality, analyses of individual dietary behaviours and exploratory multivariate methods can identify behaviour-specific relationships that may otherwise remain undetected. Integrating these approaches may offer a more comprehensive framework for understanding the relationship between dietary habits and anthropometric health in young adults [48,62]. Similarly, centrality within a network reflects the interrelationships observed among variables and should not be interpreted as demonstrating that a particular dietary behaviour causally influences the remaining behaviours [70].

The findings of the present study have important implications for university health promotion. The university years represent a critical transitional period characterised by increasing autonomy in food choices, changing daily routines, academic demands, and prolonged exposure to campus food environments. Consequently, higher education institutions provide an ideal setting for implementing preventive nutrition strategies before long-term cardiometabolic risk becomes established. Evidence from recent systematic reviews suggests that educational, environmental, and student-centred interventions implemented in higher education settings can promote healthier food choices, improve selected dietary behaviours, and contribute to addressing food insecurity among university students [71,72]. If confirmed in longitudinal and intervention studies, these findings could contribute to the development of more targeted university nutrition strategies.

The present findings further suggest that nutritional screening in university settings may benefit from combining global dietary quality assessments with targeted questions addressing specific dietary behaviours. Rather than relying exclusively on composite dietary scores, screening protocols could identify frequent sugar-sweetened beverage consumption, breakfast-related practices, and other modifiable behaviours that are more readily translated into personalised nutritional counselling. Such strategies could be integrated into student health services, campus nutrition programmes, digital health interventions, and university food-service policies. This behaviour-oriented approach is increasingly advocated within contemporary precision-nutrition and public-health nutrition frameworks [73,74,75].

The inclusion of waist circumference also highlights the value of considering measures of central adiposity alongside BMI in university health assessments whenever feasible. Although BMI remains useful for population surveillance, waist circumference and waist-to-height ratio provide complementary information beyond BMI regarding abdominal fat accumulation and cardiometabolic risk [14]. Preventive strategies should avoid weight-stigmatising approaches and instead promote supportive food environments that facilitate affordable, culturally appropriate, and sustainable healthy eating behaviours.

The present study has several important strengths. First, it evaluated a dietary behaviour questionnaire specifically developed for the Chilean population using a comprehensive psychometric framework that included internal consistency, exploratory factor analysis, parallel analysis, principal component analysis, and confirmatory modelling, providing a comprehensive assessment of the questionnaire’s measurement properties [39]. Second, the analytical strategy incorporated HC3 heteroscedasticity-consistent standard errors, bootstrap procedures, robust regression sensitivity analyses, FDR correction, and complementary exploratory techniques, including principal component analysis and network analysis. This combination allowed the robustness of the primary estimates to be assessed while providing complementary exploratory information on individual dietary behaviour [75,76]. Finally, the inclusion of waist circumference and waist-to-height ratio alongside BMI enabled a more comprehensive assessment of adiposity, while rigorous data management procedures enhanced the quality and reproducibility of the analytical dataset.

Several limitations should nevertheless be acknowledged. The cross–sectional design precludes causal inference. Participants were recruited from a single Chilean university using convenience sampling, which may have introduced selection bias because students who chose to participate could differ systematically from those who chose not to join the study. Consequently, the study sample may not be representative of the broader Chilean university student population, limiting the generalisability of the findings. Dietary behaviours were self-reported and may therefore have been affected by recall bias and social desirability bias [77]. Body-composition measures were not available for analysis, preventing assessment of adiposity phenotypes such as normal-weight obesity. Furthermore, the questionnaire assessed habitual dietary behaviours rather than quantitative dietary intake. Residual confounding is also an important limitation because several behavioural and socioeconomic factors potentially related to both dietary behaviours and adiposity, including physical activity, sedentary behaviour, sleep, psychological stress, socioeconomic status, food insecurity, and total energy intake, were not collected and therefore, could not be incorporated into the primary regression models. Consequently, the observed associations should not be interpreted as independent of these broader lifestyle and contextual factors. Finally, no formal a priori sample size calculation was performed, and no dedicated assessment of sample-size adequacy was conducted for the exploratory network analysis; therefore, the adequacy of the sample for this analysis could not be formally established. Moreover, although FDR correction and sensitivity analyses were applied, the item-level and network analyses should be interpreted as exploratory and hypothesis-generating.

Replication in multicentre samples from different regions of Chile and other geographical settings would help determine the generalisability of the observed associations across different sociocultural and food environments. Future studies using more detailed dietary assessment methods and incorporating relevant behavioural and socioeconomic factors would also provide a more complete understanding of these relationships. Prospective longitudinal studies conducted in larger and more diverse university populations are also needed to confirm these findings, establish their temporal relationships, and further evaluate the complementary value of integrating global dietary scores with individual dietary behaviour analyses.

## 5. Conclusions

In this cross-sectional sample of Chilean university students, predefined dietary behaviour scores showed limited associations with anthropometric outcomes. Although a higher Unhealthy Eating Score was nominally associated with greater waist circumference, this association did not remain statistically significant after correction for multiple comparisons, and no score-based associations were observed for BMI or waist-to-height ratio. Exploratory item-level analyses suggested associations involving sugar-sweetened beverage and breakfast consumption, although these findings require confirmation in prospective studies. Psychometric analyses provided only partial support for the predefined scoring structure, with stronger measurement properties for the healthy than for the unhealthy dietary domain. Larger, multicentre longitudinal studies incorporating more comprehensive dietary assessment, additional behavioural and lifestyle covariates, and further psychometric validation are needed to confirm these findings and determine whether item-level analyses provide complementary information beyond that obtained from composite dietary scores.

## Figures and Tables

**Table 1:** Characteristics of the study participants according to sex.

Variable	Overall (*n* = 374)	Men (*n* = 223)	Women (*n* = 151)	*p*
Age (years)	22.02 ± 2.46	21.91 ± 2.47	22.19 ± 2.45	0.273
Weight (kg)	70.87 ± 12.26	70.87 ± 11.66	70.87 ± 13.12	0.638
Height (m)	1.71 ± 0.08	1.72 ± 0.08	1.70 ± 0.09	0.010
BMI (kg/m^2^)	24.00 ± 3.05	23.80 ± 2.83	24.30 ± 3.34	0.292
Waist Circumference (cm)	78.80 ± 9.08	78.45 ± 8.50	79.31 ± 9.88	0.670
Waist-to-Height Ratio	0.46 ± 0.05	0.46 ± 0.04	0.47 ± 0.05	0.122
Healthy Eating Score	26.47 ± 5.42	26.08 ± 5.74	27.06 ± 4.87	0.063
Unhealthy Eating Score	14.47 ± 3.28	14.57 ± 3.28	14.33 ± 3.28	0.463
Overall Diet Score	12.02 ± 6.68	11.50 ± 6.88	12.79 ± 6.32	0.032

Values are presented as mean ± standard deviation unless otherwise indicated. *p*-values represent comparisons between women and men.

**Table 2:** Psychometric properties of the dietary habits questionnaire used for Chilean university students.

Domain	Statistic	Value
Internal Consistency	Cronbach’s α (overall)	0.516
	Cronbach’s α (healthy domain)	0.670
	Cronbach’s α (unhealthy domain)	0.472
	McDonald’s ω	0.323
Sampling adequacy	KMO	0.713
	Bartlett’s χ^2^	702.38
	Bartlett’s *p*	<0.001
Confirmatory Factor Analysis	CFI	0.822
	TLI	0.790
	RMSEA	0.057
	SRMR	0.064

KMO: Kaiser–Meyer–Olkin measure of sampling adequacy; CFI: Comparative Fit Index; TLI: Tucker–Lewis Index; RMSEA: Root Mean Square Error of Approximation; SRMR: Standardized Root Mean Square Residual.

**Table 3:** Associations between dietary scores and anthropometric outcomes estimated using multivariable linear regression models.

Outcome	Exposure	β (HC3)	95% CI	*p*	Adjusted R^2^
Body Mass Index	Healthy Eating Score	−0.043	−0.100 to 0.014	0.136	0.072
	Unhealthy Eating Score	0.010	−0.074 to 0.095	0.808	0.065
	Overall Diet Score	−0.030	−0.075 to 0.015	0.184	0.069
Waist Circumference	Healthy Eating Score	−0.054	−0.207 to 0.099	0.489	0.064
	Unhealthy Eating Score	0.285	0.011 to 0.560	0.041	0.073
	Overall Diet Score	−0.106	−0.238 to 0.025	0.111	0.069
Waist-to-Height Ratio	Healthy Eating Score	−0.0006	−0.0014 to 0.0001	0.109	0.093
	Unhealthy Eating Score	0.001	−0.0004 to 0.0024	0.150	0.092
	Overall Diet Score	−0.0006	−0.0013 to 0.0000	0.055	0.095

β represents the adjusted unstandardised regression coefficient, expressed as the mean difference in the anthropometric outcome associated with a one-point increase in the corresponding dietary score. Models were adjusted for age and sex. The 95% confidence intervals and *p*-values were calculated using HC3 heteroscedasticity-consistent standard errors.

## Data Availability

The data presented in this study are available on request from the corresponding author. The data are not publicly available due to ethical standards.

## Supplementary Materials

The following supporting information can be downloaded at https://www.mdpi.com/article/10.3390/healthcare14182963/s1, Table S1: Sensitivity analyses of the associations between dietary scores and anthropometric outcomes using robust regression and bootstrap confidence intervals; Table S2: Individual dietary behaviours significantly associated with anthropometric outcomes after HC3 robust regression and false discovery rate correction; Table S3: Centrality measures of individual dietary behaviours identified in the exploratory dietary behaviour network.

## Abbreviations

The following abbreviations are used in this manuscript:

BMI	Body Mass Index
CFA	Confirmatory Factor Analysis
CFI	Comparative Fit Index
CI	Confidence Interval
FDR	False Discovery Rate
HC3	Heteroscedasticity-Consistent Standard Errors (HC3 estimator)
KMO	Kaiser–Meyer–Olkin Measure of Sampling Adequacy
PCA	Principal Component Analysis
RMSEA	Root Mean Square Error of Approximation
SD	Standard Deviation
SRMR	Standardised Root Mean Square Residual
TLI	Tucker–Lewis Index
WHtR	Waist-to-Height Ratio
