# Supplementary material for: Associations of Overall Diet Quality and Individual Dietary Behaviours with Anthropometric Outcomes Among Chilean University Students
Source: Healthcare (Basel). 2026 Sep 11;14(18):2963. doi: 10.3390/healthcare14182963 (PMC13606437; doi:10.3390/healthcare14182963)
Supplement: Supplementary file 1 [file healthcare-14-02963-s001.zip › healthcare-4545763-supplementary.pdf]

**Table S1.** Sensitivity analyses of the associations between dietary scores and anthropometric outcomes using robust regression and bootstrap confidence intervals.

| Outcome | Dietary score | Robust β | Bootstrap 95% CI | Approximate p-value |
|---|---|---|---|---|
| BMI | Healthy Eating Score | -0.031 | -0.100 to 0.014 | 0.070 |
| | Unhealthy Eating Score | -0.008 | -0.072 to 0.095 | 0.418 |
| | Overall Diet Score | -0.017 | -0.076 to 0.014 | 0.086 |
| Waist circumference | Healthy Eating Score | -0.057 | -0.207 to 0.105 | 0.231 |
| | Unhealthy Eating Score | **0.312** | **0.006 to 0.555** | **0.023** |
| | Overall Diet Score | -0.115 | -0.247 to 0.024 | 0.056 |
| Waist-to-height ratio | Healthy Eating Score | -0.001 | -0.0014 to 0.0001 | 0.053 |
| | Unhealthy Eating Score | 0.001 | -0.0003 to 0.0024 | 0.069 |
| | Overall Diet Score | **-0.001** | **-0.0013 to -0.0000** | **0.025** |

Values represent regression coefficients obtained from robust regression models. Bootstrap confidence intervals were estimated using non-parametric resampling procedures. Bold values indicate statistical significance ($p < 0.05$).

**Table S2.** Individual dietary behaviours significantly associated with anthropometric outcomes after HC3 robust regression and false discovery rate correction.

| Outcome | Dietary behaviour | β (HC3) | 95% CI | p | FDR q |
|---|---|---|---|---|---|
| BMI | Breakfast consumption | -0.466 | -0.761 to -0.172 | 0.002 | 0.030 |
| Waist circumference | Sugar-sweetened beverage consumption | 1.071 | 0.425 to 1.716 | 0.001 | 0.018 |

Models were adjusted for age and sex using HC3 heteroscedasticity-consistent standard errors. P-values were corrected for multiple comparisons using the Benjamini–Hochberg false discovery rate procedure. Only associations remaining statistically significant after false discovery rate correction are presented.

**Table S3.** Centrality measures of individual dietary behaviours identified in the exploratory dietary behaviour network.

| Dietary behaviour | Community | Strength | Expected influence |
|---|---|---|---|
| H3 | 1 | 2.176 | 1.627 |
| H4 | 1 | 2.043 | 1.752 |
| U5 | 2 | 1.954 | 0.740 |
| H1 | 1 | 1.901 | 0.634 |
| H7 | 1 | 1.776 | 1.034 |
| H5 | 1 | 1.679 | 1.436 |
| U3 | 2 | 1.414 | 0.429 |
| H6 | 1 | 1.409 | 1.409 |
| H2 | 1 | 1.233 | 1.233 |
| H9 | 1 | 1.099 | 1.099 |
| U1 | 2 | 0.951 | 0.328 |
| H8 | 1 | 0.823 | 0.823 |
| U6 | 2 | 0.370 | 0.370 |
| U4 | 2 | 0.317 | 0.049 |
| U2 | 2 | 0.170 | 0.170 |

Dietary behaviour codes are as follows: H1, snack consumption; H2, low-fat dairy consumption; H3, fruit consumption; H4, vegetable consumption; H5, fish consumption; H6, legume consumption; H7, oat or whole-grain bread consumption; H8, home-cooked meal consumption; H9, dinner consumption; U1, alcoholic beverage consumption; U2, sugar-sweetened beverage consumption; U3, fried food consumption; U4, adding salt to food before tasting; U5, fast-food consumption; U6, breakfast consumption. Node strength represents the sum of the absolute edge weights connected to each dietary behaviour, whereas expected influence represents the signed sum of connected edge weights. Community membership was identified using the Clauset–Newman–Moore greedy modularity optimisation algorithm. Higher values indicate greater relative importance within the dietary behaviour network.
